# Hospitalization outcomes among brain metastasis patients receiving radiation therapy with or without stereotactic radiosurgery from the 2005–2014 Nationwide Inpatient Sample

**DOI:** 10.1038/s41598-021-98563-y

**Published:** 2021-09-28

**Authors:** Hind A. Beydoun, May A. Beydoun, Shuyan Huang, Shaker M. Eid, Alan B. Zonderman

**Affiliations:** 1 Department of Research Programs, Fort Belvoir Community Hospital, 9300 DeWitt Loop, Fort Belvoir, VA 22060 USA; 2 Laboratory of Epidemiology and Population Sciences, National Institute on Aging, NIA/NIH/IRP, Baltimore, MD USA; 3 Department of Research Programs, Fort Belvoir Community Hospital, Fort Belvoir, VA USA; 4 Department of Medicine, Johns Hopkins University School of Medicine, Baltimore, MD USA

**Keywords:** Biotechnology, Cancer, Neuroscience, Diseases, Health care, Oncology

## Abstract

The purpose of this study was to compare hospitalization outcomes among US inpatients with brain metastases who received stereotactic radiosurgery (SRS) and/or non-SRS radiation therapies without neurosurgical intervention. A cross-sectional study was conducted whereby existing data on 35,199 hospitalization records (non-SRS alone: 32,981; SRS alone: 1035; SRS + non-SRS: 1183) from 2005 to 2014 Nationwide Inpatient Sample were analyzed. Targeted maximum likelihood estimation and Super Learner algorithms were applied to estimate average treatment effects (ATE), marginal odds ratios (MOR) and causal risk ratio (CRR) for three distinct types of radiation therapy in relation to hospitalization outcomes, including length of stay (‘ ≥ 7 days’ vs. ‘ < 7 days’) and discharge destination (‘non-routine’ vs. ‘routine’), controlling for patient and hospital characteristics. Recipients of SRS alone (ATE = − 0.071, CRR = 0.88, MOR = 0.75) or SRS + non-SRS (ATE = − 0.17, CRR = 0.70, MOR = 0.50) had shorter hospitalizations as compared to recipients of non-SRS alone. Recipients of SRS alone (ATE = − 0.13, CRR = 0.78, MOR = 0.59) or SRS + non-SRS (ATE = − 0.17, CRR = 0.72, MOR = 0.51) had reduced risks of non-routine discharge as compared to recipients of non-SRS alone. Similar analyses suggested recipients of SRS alone had shorter hospitalizations and similar risk of non-routine discharge when compared to recipients of SRS + non-SRS radiation therapies. SRS alone or in combination with non-SRS therapies may reduce the risks of prolonged hospitalization and non-routine discharge among hospitalized US patients with brain metastases who underwent radiation therapy without neurosurgical intervention.

## Introduction

Brain metastases are the most common intracranial tumors affecting 8–10% of cancer patients; the majority experience 1–3 brain lesions with poor prognosis in terms of overall survival, progression-free survival and neurological function^1–4^. Uncontrolled brain metastases often result in headaches, neurocognitive dysfunction, seizures, and eventually death^3^. Approximately 170,000 cases are diagnosed on an annual basis in the United States^5,6^, and the frequency is expected to increase with improved therapeutic options and continued routine surveillance using magnetic resonance imaging^3^.

Although brain metastases are the main cause of morbidity and mortality among cancer patients, younger age, higher Karnofsky Performance Score (KPS), fewer brain lesions, and uncontrolled extracranial disease were associated with improved prognosis^5^. The life expectancy of patients having brain metastases is approximately 12 months if they receive recommended radiation and/or surgical therapies or less than 12 months if they only receive supportive care^1,7^. Whereas neurologic manifestations affect 70% of patients with brain metastases potentially reducing their quality of life, recommended treatments (whole brain radiotherapy (WBRT), stereotactic radiosurgery (SRS) and/or surgical resection) may incur considerable costs on the healthcare system with varying impact on neurological function^1,7^.

Treatment selection depends on multiple health-related criteria with overarching goals of achieving local tumor control, improving quality of life and preventing death from neurological disease^2^. In general, surgical resection is preferred for patients with single, operable and/or large brain metastases causing edema or hydrocephalus. By contrast, WBRT is useful as adjuvant therapy post-surgical resection or for multiple brain metastases that cannot be surgically removed^2,4^. WBRT alone is indicated among patients with poor performance or > 3 brain metastases^4^. As defined by the American Association of Neurological Surgeons (AANS) and American Society for Therapeutic Radiology and Oncology (ASTRO), SRS is “a distinct neurosurgical discipline that utilizes externally generated ionizing radiation to inactivate or eradicate defined targets in the head or spine without the need to make an incision”^2^. Patients having 1–3 brain metastases or good performance based on KPS often receive a combination of WBRT and SRS^4^, and those refusing WBRT or needing salvage treatment post-WBRT often receive SRS alone^4^.

SRS allows precise delivery of high-dose radiation in a single or up to five sessions to a specific target in the brain, using an accelerator under the guidance of real-time imaging, whereas WBRT often necessitates multiple sessions of radiation therapy^2,3,7,8^. Nevertheless, the optimal choice of radiation therapy for brain metastases remains unclear given that SRS focuses on visible brain metastases with possible failure to address microscopic metastases or metastases found in other regions of the brain^3,7^. By contrast, WBRT may optimize tumor control with diminished need for salvage treatment while being linked to worse neurocognitive function^3,7^. In recent years, SRS alone has been shifted to the outpatient setting with the more complex cases being treated in an inpatient setting, whereas SRS as an adjunctive treatment remains stable among hospitalized patients^9^. Since SRS is considerably more expensive than WBRT, healthcare resource utilization outcomes in the context of patients receiving SRS and/or non-SRS radiation therapies for brain metastases require further elucidation, especially in the context of hospitalizations^10^.

The purpose of this cross-sectional study is to compare hospitalization outcomes among US patients with brain metastases who received SRS and/or non-SRS radiation therapies without neurosurgical intervention. We hypothesized that SRS ± non-SRS therapies will be more expensive but will achieve better clinical outcomes at discharge compared to non-SRS therapies alone controlling for patient and hospital characteristics using targeted maximum likelihood estimation (TMLE) and Super Learner algorithms.

## Methods

### Data source

The Agency for Healthcare Research and Quality (AHRQ) Healthcare Cost and Utilization Project (HCUP) Nationwide Inpatient Sample (NIS) is the largest publicly available, all-payer inpatient care database of community hospitals in the United States. It consists of ~ 5–8 million discharge records sampled annually from ~ 1000 hospitals since 1988. Each year, ~ 20% stratified probability sample of hospitals (before 2012) or discharge records (since 2012) is selected from all participating HCUP states. NIS data elements included patient demographics, ~ 15 diagnoses, ~ 15 procedures as well as hospital course and outcomes. Since the project was determined to be research not involving human subjects, a waiver of institutional review board approval was granted at Fort Belvoir Community Hospital. Due to the nature of the research study, informed consent was not needed as determined at Fort Belvoir Community Hospital. The project adhered to relevant ethical guidelines/regulations in accordance with the Declaration of Helsinki.

### Study participants

The population consisted of discharge records from the 2005–2014 NIS databases that met the following inclusion criteria: (1) Primary or secondary ICD-9-CM diagnosis of brain metastases (191 *(malignant neoplasm brain)*, 191.7 *(malignant neo brain stem)*, 191.8 *(malignant neo brain nec)*, 191.9 *(malignant neo brain nos)*, 198.3 *(sec mal neo brain/spine)*) based on up to 15 diagnostic codes; (2) Non-SRS (92.2 (therap radiol & nucl med*), 92.21 *(superficial radiation)*, 92.22 *(orthovoltage radiation)*, 92.23 *(radioisot teleradiother)*, 92.24 *(teleradio using photons)*, 92.25 *(electron teleradiotherap)*, 92.26 *(particul teleradioth nec)*, 92.27*(radioactive elem implant)*, 92.29 *(radiotherapeut proc nec)*, 92.31 *(sing source radiosurgery)*, 92.32 *(multisource radiosurgery)*, 92.33 *(particulate radiosurgery)*) and/or SRS (92.3 (stereotact radiosurgery*), 92.30 *(stereo radiosurgery nos)*, 92.39 *(stereo radiosurgery nec)*) radiation therapies based on up to 15 procedure codes. Discharge records were excluded for patients who: (1) had simultaneously undergone neurosurgical procedures (01 (incise-excis brain/skull*); 01.5 (excise brain & meninges*); 01.59 *(other brain excision)*; 01.6 *(excise skull lesion)*; 03.4 *(excis spinal cord lesion)*; 04.0 (per nerv incis/div/excis*); 04.01 *(excision acoustc neuroma)*; 04.07 *(periph nerv excision nec)*; or (2) had missing data on patient/hospital characteristics and/or hospitalization outcomes.

### Study variables

Outcome variables of interest were length of stay and discharge destination. Length of stay (days) was dichotomized as ‘ ≥ 7 days’ vs. ‘ < 7 days’. Discharge destination was defined as ‘non-routine’ (discharged to an institution or died while hospitalized) vs. ‘routine’ (discharged home), after exclusion of records for patients discharged alive with unknown destination. Exposure variables were defined for comparing types of radiation therapy: [1] ‘SRS alone’ vs. ‘non-SRS alone’, [2] ‘SRS + non-SRS’ vs. ‘non-SRS alone’ and [3] ‘SRS alone’ vs. ‘SRS + non-SRS’. Patient characteristics were defined as sex, age, race/ethnicity, Charlson comorbidity index (CCI), admission type, admission quarter, weekend admission, primary health insurance and number of procedures whereas hospital characteristics were defined as region, location & teaching status and bed size.

### Statistical analysis

All statistical analyses were performed using Stata release 16 (StataCorp19. Stata Statistical Software; Release 16. College Station, TX: StataCorp LLC) while considering complex sampling design. We applied uncorrected Chi-square, design-based F-tests and regression modeling to examine bivariate associations. First, logistic regression models were constructed to estimate odds ratios (OR) and 95% confidence intervals (CI) for each patient/hospital characteristic as predictor for radiation therapy type, taking non-SRS alone as a referent. Second, linear and logistic regression models were constructed to estimate β coefficients and OR with their 95% CI for distinct types of radiation therapy as predictors of continuous and dichotomous outcomes, respectively. Finally, Ensemble Learning TMLE was performed to estimate the average treatment effects (ATE) of dichotomous exposure (‘SRS alone’ vs. ‘non-SRS alone’; ‘SRS + non-SRS’ vs. ‘SRS alone’; ‘SRS alone’ vs. ‘SRS + non-SRS’) on length of stay (‘ ≥ 7 days’ vs. ‘ < 7 days’) and discharge destination (‘non-routine’ vs. ‘routine’), using *eltmle* package in Stata. Because regression methods are frequently biased if outcome models are misspecified, methods incorporating propensity scores, the G-formula or TMLE are preferred^11^. While propensity score methods necessitate exposure models to be correctly specified, double-robust methods such as TMLE require correct specification of either outcome or exposure models^11^. TMLE is a semiparametric estimator allowing use of machine learning algorithms to minimize model misspecification^11^. Unlike TMLE, classical regression methods for estimating ATE, or risk difference, assume that ATE is constant across confounder levels with no effect modification. We used Super Learner with tenfold cross-validation to evaluate predictive performance for potential outcomes and weighted averages as a propensity score for distinct machine learning algorithms. The default Super Learner machine learning algorithm was applied as previously defined in an R v.1.2.0-5 package as follows: [1] stepwise selection, [2] generalized linear modeling (GLM), [3] GLM variant that includes second order polynomials and two-by-two interactions of main terms included in the model. The ATE, causal risk ratio (CRR) and marginal odds ratio (MOR) were estimated with 95% CI for each hypothesized relationship using TMLE^11–15^. We performed two-sided statistical tests whereby p < 0.05 was considered statistically significant.

### Ethical approval

Since the project was determined to be research not involving human subjects, a waiver of institutional review board approval was granted at Fort Belvoir Community Hospital. Due to the nature of the research study, informed consent was not needed as determined at Fort Belvoir Community Hospital. The project adhered to relevant ethical guidelines/regulations in accordance with the Declaration of Helsinki.

### Disclaimer

The views expressed in this article are those of the authors and do not reflect the official policy of Fort Belvoir Community Hospital, the Defense Health Agency, Department of Defense, or the US Government. Any discussion or mention of commercial products or brand names does not imply or support any endorsement by the Federal Government.

## Results

**Figure 1:**
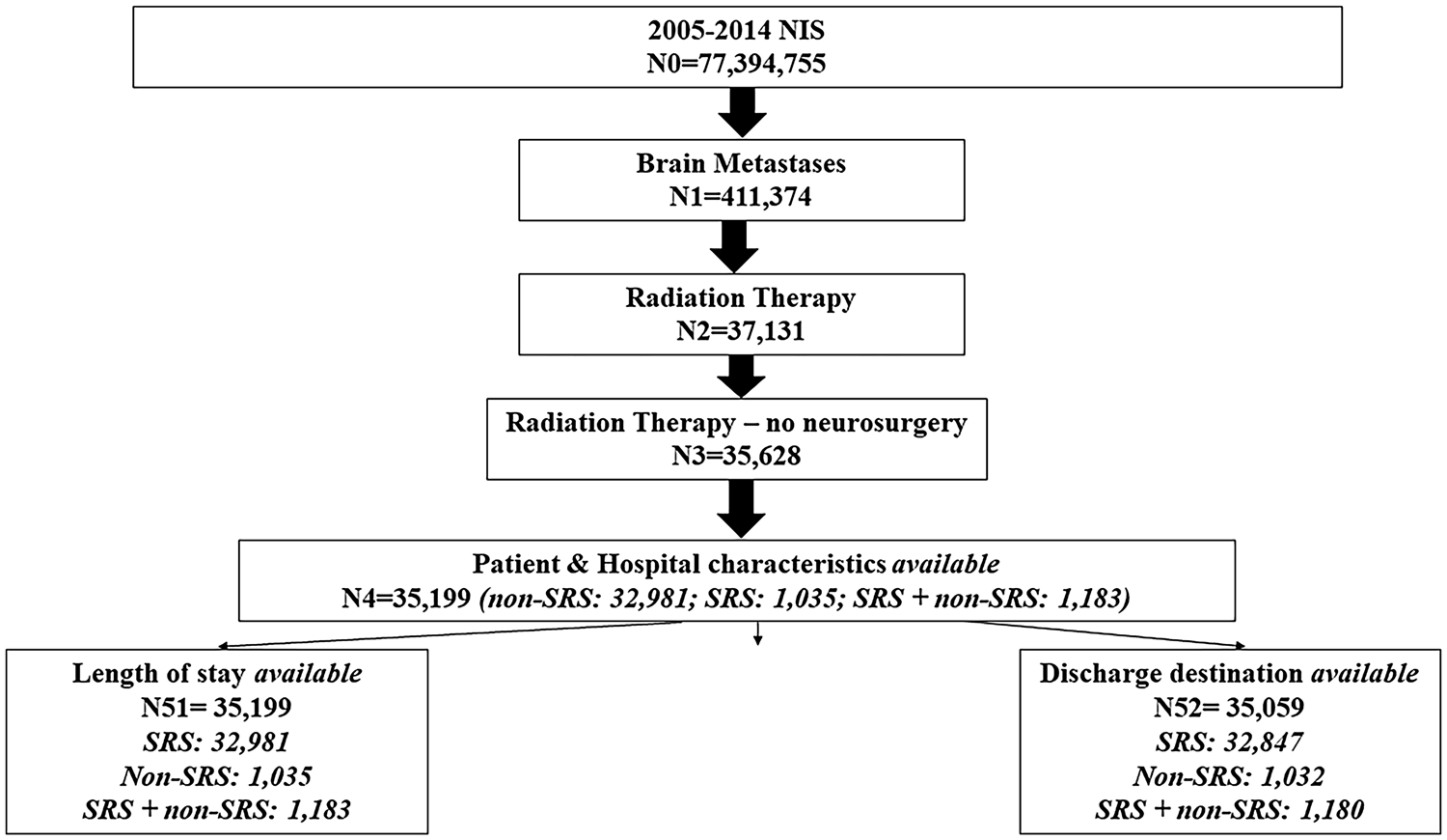
Study flowchart—Nationwide Inpatient Sample (2005–2014).

Among 77,394,755 hospitalization records identified from the 2005–2014 NIS database, 411,374 corresponded to patients with primary or secondary diagnosis of brain metastasis. Of those, 37,131 corresponded to patients who received radiation therapy with or without neurosurgery. A total of 35,628 records remained after excluding patients who underwent neurosurgery. The final analytic sample consisted of 35,199 hospitalization records (non-SRS alone: 32,981; SRS alone: 1035; SRS + non-SRS: 1183) with non-missing patient/hospital characteristics data; of those, 35,199 had non-missing data on length of stay and 35,059 had non-missing data on discharge destination (Fig. 1).

A large percentage of records corresponded to patients who reported being 50–79 years of age (75.3%), of White race (61.7%), Medicare recipients (43.9%), being admitted electively (83.9%), on weekdays (81.7%), to Western (37.3%), urban (teaching) (66.0%) and/or large (73.6%) hospitals. Recipients of non-SRS alone, SRS alone and SRS + non-SRS differed on most patient/hospital characteristics. Compared to recipients of non-SRS therapies alone, those who received SRS alone were less likely to be female, ≥ 50 years, African Americans, to have CCI ≥ 7 and/or to be hospitalized on weekends at Midwestern/Western hospitals. Patients who underwent SRS alone were more frequently 20–29 years of age, recipients of private health insurance and/or admitted on an emergency basis, to large, urban hospitals compared to those who underwent non-SRS therapies alone. Recipients of SRS + non-SRS therapies were more frequently ≥ 20 years of age, Hispanic, admitted to Southern hospitals on an emergency basis and/or more likely to receive 5+ procedures. These patients were less frequently African-Americans, with CCI ≥ 7, admitted on weekends to medium/large urban, non-teaching hospitals and/or recipients of non-Medicare/non-private health insurance compared to those who underwent non-SRS therapies alone (Table 1).

**Table 1:** Recipients of non-SRS alone, SRS alone and SRS + non-SRS for the treatment of brain metastases by patient and hospital characteristics—2005–2014 Nationwide Inpatient Sample (n = 35,199).

	Total	Non-SRS alone^a^	SRS alone		SRS + non-SRS	
	%	%	%	OR (95% CI)	%	OR (95% CI)
**Overall**						
Sex						
Male	47.3	47.0	52.4	Ref.	47.9	Ref.
Female	52.7	52.9	47.5	0.80 (0.71, 0.91)	52.1	0.96 (0.86, 1.08)
Age (years)						
< 20	2.1	2.1	3.4	Ref.	0.9	Ref.
20–29	1.1	1.1	2.9	1.68 (1.01, 2.78)	16.2	3.36 (1.58, 7.16)
30–39	3.1	3.0	5.7	1.15 (0.75, 1.78)	3.8	2.73 (1.40, 5.33)
40–49	10.7	10.6	14.1	0.83 (0.57, 1.21)	11.4	2.38 (1.28, 4.44)
50–59	25.1	25.2	25.7	0.63 (0.44, 0.91)	23.8	2.08 (1.13, 3.83)
60–69	29.4	29.4	27.9	0.59 (0.41, 0.84)	30.4	2.29 (1.25, 4.19)
70–79	20.8	20.9	15.4	0.45 (0.31, 0.66)	20.8	2.19 (1.19, 4.03)
≥ 80	7.4	7.5	4.8	0.39 (0.25, 0.62)	7.3	2.15 (1.14, 4.05)
Race/ethnicity						
White	61.7	61.4	73.7	Ref.	59.2	Ref.
African American	12.3	12.6	6.8	0.44 (0.35, 0.57)	7.2	0.58 (0.47, 0.74)
Hispanic	5.4	5.3	5.9	0.93 (0.71, 1.22)	8.9	1.74 (1.42, 2.14)
Other	4.6	4.7	5.4	0.96 (0.72, 1.26)	3.9	0.88 (0.65, 1.19)
Unknown	15.9	15.9	8.3	0.43 (0.34, 0.54)	20.7	1.34 (1.17, 1.55)
Charlson comorbidity index						
2–6	53.5	52.7	72.9	Ref.	60.9	Ref.
≥ 7	46.4	47.2	27.1	0.41 (0.36, 0.48)	39.0	0.71 (0.63, 0.80)
Admission type						
Elective	83.9	85.8	51.5	Ref.	60.1	Ref.
Emergency	16.0	14.2	48.4	5.68 (5.00, 6.43)	39.8	3.99 (3.55, 4.49)
Admission quarter						
1st quarter	25.8	25.7	26.5	Ref.	27.3	Ref.
2nd quarter	25.2	25.2	27.2	1.05 (0.88, 1.24)	24.4	0.91 (0.77, 1.07)
3rd quarter	24.6	24.7	22.6	0.88 (0.74, 1.06)	24.3	0.93 (0.79, 1.09)
4th quarter	24.3	24.4	23.5	0.94 (0.79, 1.12)	23.9	0.93 (0.78, 1.09)
Weekend admission						
Weekday	81.7	81.3	88.3	Ref.	86.9	Ref.
Weekend	18.3	18.7	11.6	0.57 (0.47, 0.69)	13.1	0.65 (0.55, 0.78)
Primary health insurance						
Medicare	43.9	44.0	35.6	Ref.	46.6	Ref.
Medicaid	14.2	14.4	12.9	1.11 (.91, 1.36)	10.1	0.66 (0.54, 0.82)
Private	35.9	35.5	46.3	1.62 (1.41, 1.86)	39.2	1.04 (0.92, 1.19)
Self-pay/no pay/other	5.9	6.0	5.1	1.04 (0.78, 1.40)	3.9	0.62 (0.45, 0.83)
Number of procedures						
0–4	84.4	84.4	85.8	Ref.	81.2	Ref.
≥ 5	15.6	15.5	14.1	0.89 (0.75, 1.07)	18.7	1.25 (1.08, 1.45)
Hospital region						
Northeast	26.7	26.5	31.8	Ref.	25.4	Ref.
Midwest	23.2	23.4	17.6	0.63 (0.52, 0.76)	23.0	1.03 (0.86, 1.22)
West	37.3	37.4	35.3	0.78 (0.68, 0.92)	34.9	0.98 (0.84, 1.13)
South	12.8	12.6	15.3	1.01 (0.83, 1.23)	16.6	1.38 (1.15, 1.64)
Hospital location/teaching status						
Rural	5.6	5.7	0.8	Ref.	5.5	Ref.
Urban, non-teaching	28.3	28.9	20.8	5.03 (2.56, 9.88)	16.3	0.58 (0.44, 0.77)
Urban, teaching	66.0	65.2	78.3	8.40 (4.32, 16.35)	78.1	1.24 (0.97, 1.59)
Bed size						
Small	8.4	8.2	6.2	Ref.	14.3	Ref.
Medium	18.0	18.0	15.0	1.10 (0.82, 1.47)	20.6	0.65 (0.55, 0.79)
Large	73.6	73.7	78.8	1.41 (1.09, 1.83)	65.0	0.50 (0.43, 0.58)

*CI* confidence intervals, *OR* odds ratio, *SRS* stereotactic radiosurgery.

^a^Reference.

Hospitalization outcomes were summarized according to radiation therapy type in Table 2. The mean and median length of stay were 9.8 days and 7 days, respectively, and 58.3% experienced non-routine hospital discharge. Recipients of non-SRS therapies experienced the longest hospitalizations and were more likely to be discharged on a non-routine basis than their counterparts. Conversely, length of hospital stay was 3–4 days shorter in the context of SRS ± non-SRS therapies and recipients of SRS ± non-SRS therapies were 3–4 times less likely to be discharged non-routinely.

**Table 2:** Recipients of non-SRS alone, SRS alone and SRS + non-SRS for the treatment of brain metastases by hospitalization outcomes—2005–2014 Nationwide Inpatient Sample.

	Total	Non-SRS alone*	SRS alone		SRS + non-SRS	
**Length of stay (days) (N = 35,199)**						
Mean (95% CI)	9.81 (9.71, 9.91)	10.02 (9.93, 10.13)	6.27 (5.85, 6.69)	< 0.001	6.84 (6.38, 7.31)	< 0.001
Median (IQR)	7 (4, 13)	7 (4, 13)	4 (2, 9)		4 (1, 9)	
β (95% CI)		Ref	-3.76 (-4.20, -3.31)		-3.18 (-3.66, -2.70)	
< 7, %	44.0	43.3	64.0	< 0.001	63.4	< 0.001
≥ 7, %	55.0	56.7	35.9		36.4	
OR (95% CI)		Ref	0.43 (0.37, 0.48)		0.44 (0.39, 0.49)	
**Discharge destination (N = 35,059)**						
Routine, %	41.6	40.1	65.8	< 0.001	61.8	< 0.001
Non-routine, %	58.3	59.8	34.2		38.2	
Short-term hospital, %	1.5	1.6	0.7		1.2	
Transferred to SNF / ICF / other, %	25.6	25.9	19.9		20.2	
AMA, %	25.1	25.8	13.0		15.1	
Died, %	6.0	6.3	0.6		1.7	
OR (95% CI)		Ref	0.34 (0.31, 0.39)		0.42 (0.37, 0.47)	

*AMA* against medical advice, *CI* confidence intervals, *IQR* interquartile range, *ICF* intermediate care facility, *OR* odds ratio, *SNF* skilled nursing facility, *SRS* stereotactic radiosurgery.

* Reference category.

Table 3 presents results of TMLE analyses for radiation therapy types in relation to hospitalization outcomes. After applying Super Learner algorithms to calculate ATE, CRR and MOR with their 95% CI, recipients of SRS alone had shorter hospitalizations (ATE = − 0.071, CRR = 0.88, MOR = 0.75) with reduced risks of non-routine discharge (ATE = − 0.13, CRR = 0.78, MOR = 0.59) compared to non-SRS alone. Recipients of SRS + non-SRS therapies had shorter hospitalizations (ATE = − 0.17, CRR = 0.70, MOR = 0.50) and reduced risks of non-routine discharge (ATE = − 0.17, CRR = 0.72, MOR = 0.51) compared to recipients of non-SRS alone. Similar analyses suggested recipients of SRS alone had shorter hospitalizations and similar risk of non-routine discharge compared to SRS + non-SRS therapies.

**Table 3:** Targeted maximum likelihood estimation of average treatment effects for SRS alone versus non-SRS alone, SRS + non-SRS versus non-SRS alone, SRS alone versus SRS + non-SRS) as predictors of hospitalization outcomes among US inpatients with brain metastases—2005–2014 Nationwide Inpatient Sample.

	SRS alone vs. non-SRS alone		SRS + non-SRS vs. non-SRS alone		SRS alone vs. SRS + non-SRS	
	Length of hospital stay	Discharge destination	Length of hospital stay	Discharge destination	Length of hospital stay	Discharge destination
**POM1**						
N	34,014	33,877	34,162	34,025	2218	2212
Mean (SD)	0.4964667 (0.1955594)	0.4636692 (0.1893866)	0.3969059 (0.2038177)	0.4275475 (0.1658896)	0.3361536 (0.261995)	0.3501691 (0.2169594)
Range	0.0558548, 0.9379832	0.0384601, 0.8993036	0.0607201, 0.9140593	0.041389, 0.8880401	0.0300083, 0.9429044	0.0287489, 0.8881696
**POM0**						
N	34,014	33,877	34,162	34,025	2218	2212
Mean (SD)	0.5678285 (0.138681)	0.5961083 (0.1379968)	0.5681876 (0.1394296)	0.5970576 (0.137947)	0.3964239 (0.2782597)	0.3822387 (0.2270246)
Range	0.2432608, 0.9396189	0.1400895, 0.9093559	0.2414657, 0.9379365	0.137566, 0.9120114	0.0385375, 0.9605096	0.0329898, 0.9091607
**PS**						
N	34,014	33,877	34,162	34,025	2218	2212
Mean (SD)	0.0382209 (0.0439592)	0.0382623 (0.0440574)	0.0402479 (0.0427544)	0.0402791 (0.0425867)	0.5333634 (0.17479)	0.5334539 (0.1694388)
Range	0.025, 0.4970353	0.025, 0.4998833	0.025, 0.6480741	.025, 0.6482674	0.1318027, 0.9576377	0.1452669, 0.9506442
**ATE**						
Estimate	− 0.0714	− 0.1324	− 0.1713	− 0.1695	− 0.0603	− 0.0321
SE	0.0121	0.0119	0.0126	0.0131	0.0178	0.0186
P value	0.0000	0.0000	0.0000	0.0000	0.0026	0.1813
95% CI	− 0.0951,− 0.0476	− 0.1558, − 0.1091	− 0.1959, − 0.1466	− 0.1952, − 0.1438	− 0.0952, − 0.0254	− 0.0686, 0.0044
TMLE CRR (95% CI)	0.88 (0.84, 0.92)	0.78 (0.74, 0.82)	0.70 (0.66, 0.74)	0.72 (0.68, 0.76)	0.85 (0.76, 0.95)	0.92 (0.82, 1.02)
TMLE MOR (95% CI)	0.75 (0.68, 0.83)	0.59 (0.53, 0.64)	0.50 (0.45, 0.55)	0.51 (0.45, 0.56)	0.77 (0.66, 0.89)	0.87 (0.73, 1.01)

*ATE* average treatment effect, *CI* confidence intervals, *CRR* causal risk ratio, *MOR* marginal odds ratio, *POM1* potential outcome among the treated, *POM0* potential outcome among the non-treated, *PS* propensity score, *SRS* stereotactic radiosurgery, *TMLE* targeted maximum likelihood estimation.

## Discussion

In this cross-sectional study of hospitalization records from the 2005–2014 AHRQ HCUP NIS database, we applied TMLE and Super Learner algorithms to compare distinct types of radiation therapies (SRS and/or non-SRS) on length of stay and discharge destination among US inpatients diagnosed with brain metastases. To date, SRS utilization and its outcomes have rarely been examined using the NIS—a nationally representative sample of hospitalized US patients—and most previously published studies were not focused on the treatment of brain metastases^9,16–20^. Study results suggested disparities in treatment selection according to patient and hospital characteristics, which did not fully explain the observed treatment outcome differences. Specifically, recipients of SRS alone or SRS + non-SRS therapies were more frequently admitted on an emergency basis to teaching hospitals, whereas patients who received SRS alone were often younger, with fewer comorbidities and more likely to have Medicare or private insurance. Whereas SRS alone and SRS + non-SRS therapies were less frequent among African Americans, SRS + non-SRS therapies were more frequent among Hispanics. Compared to those patients who received SRS alone or SRS + non-SRS therapies, recipients of non-SRS therapies alone were at higher risk for prolonged hospitalization and non-routine discharge. In addition, patients who underwent SRS alone had lower rates for prolonged hospitalization and similar rates of non-routine discharge as compared to those who underwent SRS + non-SRS therapies.

Whereas the indication for neurosurgical treatment is clear-cut, the choice between SRS alone or in combination with non-SRS therapies remains controversial given quality of life and cost considerations^10^. Recent studies have examined the cost-effectiveness of SRS among patients with brain metastases^3,4,7,8,21–25^. Despite evidence supporting the cost-effectiveness of SRS, previous studies have also suggested that only a small percentage of eligible US patients were selected to undergo this procedure, with clear disparities according patient and hospital characteristics, as suggested in this study. In one study, secondary analyses of existing SEER-Medicare data were performed on 7,684 elderly patients who were diagnosed with brain metastases resulting from non-small cell lung cancer (NSCLC) and treated with radiation therapy between 2000 and 2007 within two months of their diagnosis^26^. Whereas 469 (6.1%) patients had billing codes for SRS, characteristics that predicted SRS utilization included year of diagnosis, SEER registry, higher socioeconomic status, admission to a teaching hospital, no participation in low-income state buy-in programs, no extracranial metastases, and longer interval from NSCLC diagnosis^26^. Another study involving 2312 patients (813 SRS and 1499 non-SRS) from 2005 to 2014 National Cancer Database (NCD) evaluated utilization of intracranial radiotherapy for renal cell carcinoma^27^. SRS utilization increased from 27% in 2005 to 44% in 2014, and was more often reported among individuals whose place of residence was away from the facility, those who were treated at academic centers and/or those who had chemotherapy or nephrectomy^27^. By contrast, SRS was less common among individuals with lower income and those who were uninsured/had Medicaid^27^. Finally, 11,000 hospital discharge records from the 1998–2011 NIS database corresponding to patients who underwent primary or adjuvant SRS were analyzed to examine SRS trends and outcomes (in-hospital complications, mortality and resource utilization)^9^. Their results suggested that the most frequent indication for SRS remained brain metastasis (36.7%), with the complexity and severity of illness increasing over time as SRS became increasingly less frequent as a primary treatment but remained stable as an adjuvant treatment among hospitalized patients^9^. In recent years, those who received SRS while hospitalized were mostly high-risk patients who were more likely to experience poor outcomes in terms of mortality and resource utilization whereas stable patients often received SRS at outpatient centers^9^.

A causal link between treatment selection and hospitalization outcomes is ideally established in the context of randomized controlled trials, whereby random allocation of patients to various treatments aids in balancing both measured and unmeasured confounders at baseline. Statistical techniques such as TMLE are useful for dealing with confounding bias in the context of observational studies that aim to approximate randomized controlled trials. Current evidence suggests that selection of SRS and/or non-SRS therapies may be influenced by a wide range of prognostic factors, which in turn may influence healthcare utilization outcomes of hospitalized US patients diagnosed with brain metastases. For instance, patients undergoing SRS often have lower intracranial disease burden, receive fewer treatments and are more likely to be discharged routinely and earlier than patients who receive WBRT which can take 5–10 fractions and is more frequently used among patients with more extensive disease and poorer performance. Therefore, the observed associations between treatment selection and hospitalization outcomes may or may not be causal in nature since they could be explained, in part, by unmeasured prognostic factors that are amenable to SRS selection rather than to SRS itself. In this observational study, we applied TMLE, a double-robust semiparametric estimator which is superior to propensity scoring methodology in terms of disentangling the effects of treatment from those of prognostic factors. To our knowledge, this study is the first to apply Super Learner algorithms while estimating ATE using TMLE among hospitalized US patients who underwent SRS and/or non-SRS therapies for brain metastases. It has already been established that SRS is two to sixfold more expensive than non-SRS therapies^10^. However, additional research is needed to elucidate shorter hospital stays and fewer non-routine discharges among patients who underwent SRS with or without non-SRS therapies, although fewer neurological complications may be partly responsible for improved clinical outcomes among individuals treated with SRS.

Study results should be interpreted with caution in light of several limitations. First, we relied on an administrative database, which has limited scope and granularity. Unlike the SEER-Medicare and NCD databases, the NIS database does not collect detailed information on cancer diagnosis, staging and treatment. Second, complete subject analysis was performed with the potential for selection bias because of missing data. Third, many study variables, including cancer diagnosis and treatment, were defined using ICD-9 codes, potentially leading to misclassification bias. Fourth, residual confounding could not be ruled out as an alternative explanation given the observational study design and the limited availability of data elements within the NIS database, including intracranial disease burden and patient performance which can influence SRS treatment selection. Similarly, the role of chance could not be ruled out given the limited number of patients who underwent SRS during their hospital stays. Fifth, the cross-sectional design does not allow the establishment of temporality between variables of interest. Finally, study results could only be generalized to hospitalized patients within the period of interest. The demographic, socioeconomic and health characteristics of hospitalized patients may differ from those who sought outpatient care for SRS and/or non-SRS therapies at later time points.

## Conclusions

SRS alone or in combination with non-SRS therapies may reduce the risks of prolonged hospitalization and non-routine discharge among hospitalized US patients with brain metastases who underwent radiation therapy without neurosurgical intervention. In this cross-sectional study, we estimated ATE using TMLE, a double-robust semiparametric estimator that allows the use of machine learning algorithms to minimize model misspecification. Our findings are consistent with previously conducted studies that relied on national registries and databases. Prospective cohort studies are necessary to confirm and extend these preliminary findings.

## Data Availability

The data that support the findings of this study are available from AHRQ but restrictions apply to the availability of these data, which were used under license for the current study, and so are not publicly available. Specifically, the AHRQ HCUP databases were purchased by the researchers. Furthermore, AHRQ requires researchers wishing to analyze their HCUP data to complete the required training and to sign and submit a data use agreement. Data are however available from the authors upon reasonable request and with permission of AHRQ.

## References

[1] Habibi A (2018). Early palliative care for patients with brain metastases decreases inpatient admissions and need for imaging studies. Am. J. Hosp. Palliat. Care.

[2] Liu Q, Tong X, Wang J (2019). Management of brain metastases: History and the present. Chin. Neurosurg. J..

[3] Savitz ST, Chen RC, Sher DJ (2015). Cost-effectiveness analysis of neurocognitive-sparing treatments for brain metastases. Cancer.

[4] Sperduto PW (2003). A review of stereotactic radiosurgery in the management of brain metastases. Technol. Cancer Res. Treat..

[5] Kimmell KT, LaSota E, Weil RJ, Marko NF (2015). Comparative effectiveness analysis of treatment options for single brain metastasis. World Neurosurg..

[6] Miller JA (2016). Association between radiation necrosis and tumor biology after stereotactic radiosurgery for brain metastasis. Int. J. Radiat. Oncol. Biol. Phys..

[7] Kim H, Rajagopalan MS, Beriwal S, Smith KJ (2017). Cost-effectiveness analysis of stereotactic radiosurgery alone versus stereotactic radiosurgery with upfront whole brain radiation therapy for brain metastases. Clin. Oncol..

[8] Lester-Coll NH, Sher DJ (2017). Cost-Effectiveness of stereotactic radiosurgery and stereotactic body radiation therapy: A critical review. Curr. Oncol. Rep..

[9] Ho AL (2016). National trends in inpatient admissions following stereotactic radiosurgery and the in-hospital patient outcomes in the United States from 1998 to 2011. J. Radiosurg. SBRT.

[10] Shenker RF (2019). Analysis of the drivers of cost of management when patients with brain metastases are treated with upfront radiosurgery. Clin. Neurol. Neurosurg..

[11] Luque-Fernandez MA, Schomaker M, Rachet B, Schnitzer ME (2018). Targeted maximum likelihood estimation for a binary treatment: A tutorial. Stat. Med..

[12] van der Laan MJ, Polley EC, Hubbard AE (2007). Super learner. Stat. Appl. Genet. Mol. Biol..

[13] Pirracchio R, Petersen ML, van der Laan M (2015). Improving propensity score estimators' robustness to model misspecification using super learner. Am. J. Epidemiol..

[14] Wyss R (2018). Using super learner prediction modeling to improve high-dimensional propensity score estimation. Epidemiology.

[15] Ju C (2019). Propensity score prediction for electronic healthcare databases using super learner and high-dimensional propensity score methods. J. Appl. Stat..

[16] Lad SP, Santarelli JG, Patil CG, Steinberg GK, Boakye M (2009). National trends in spinal arteriovenous malformations. Neurosurg. Focus.

[17] McClelland S, Guo H, Okuyemi KS (2011). Morbidity and mortality following acoustic neuroma excision in the United States: Analysis of racial disparities during a decade in the radiosurgery era. Neuro Oncol..

[18] Patel S (2013). Trends in surgical use and associated patient outcomes in the treatment of acoustic neuroma. World Neurosurg..

[19] Wang DD (2013). Trends in surgical treatment for trigeminal neuralgia in the United States of America from 1988 to 2008. J. Clin. Neurosci..

[20] McClelland S, Jalai CM, Ryu S, Passias PG (2016). Limitations of using population-based databases to assess trends in spinal stereotactic radiosurgery. J. Radiosurg..

[21] Bijlani A, Aguzzi G, Schaal DW, Romanelli P (2013). Stereotactic radiosurgery and stereotactic body radiation therapy cost-effectiveness results. Front. Oncol..

[22] Lal LS (2011). Economic impact of stereotactic radiosurgery for malignant intracranial brain tumors. Expert Rev. Pharmacoecon. Outcomes Res..

[23] Lam TC, Sahgal A, Chang EL, Lo SS (2014). Stereotactic radiosurgery for multiple brain metastases. Expert Rev. Anticancer Ther..

[24] Lester-Coll NH (2016). Cost-effectiveness of stereotactic radiosurgery versus whole-brain radiation therapy for up to 10 brain metastases. J. Neurosurg..

[25] Warsi NM (2020). The role of stereotactic radiosurgery in the management of brain metastases from a health-economic perspective: A systematic review. Neurosurgery.

[26] Halasz LM, Weeks JC, Neville BA, Taback N, Punglia RS (2013). Use of stereotactic radiosurgery for brain metastases from non-small cell lung cancer in the United States. Int. J. Radiat. Oncol. Biol. Phys..

[27] Haque W, Verma V, Butler EB, Teh BS (2018). Utilization of stereotactic radiosurgery for renal cell carcinoma brain metastases. Clin. Genitourin. Cancer.

